# The description–experience gap: a challenge for the neuroeconomics of decision-making under uncertainty

**DOI:** 10.1098/rstb.2019.0665

**Published:** 2021-01-11

**Authors:** Basile Garcia, Fabien Cerrotti, Stefano Palminteri

**Affiliations:** Laboratoire de Neurosciences Cognitives et Computationnelles, Ecole Normale Supérieure, Institut National de la Santé et Recherche Médicale, Université de Recherche Paris Sciences et Lettres, Paris, France

**Keywords:** neuroeconomics, description–experience gap, reinforcement learning, decision-making, macaque, risk

## Abstract

The experimental investigation of decision-making in humans relies on two distinct types of paradigms, involving either description- or experience-based choices. In description-based paradigms, decision variables (i.e. payoffs and probabilities) are explicitly communicated by means of symbols. In experience-based paradigms decision variables are learnt from trial-by-trial feedback. In the decision-making literature, ‘description–experience gap’ refers to the fact that different biases are observed in the two experimental paradigms. Remarkably, well-documented biases of description-based choices, such as under-weighting of rare events and loss aversion, do not apply to experience-based decisions. Here, we argue that the description–experience gap represents a major challenge, not only to current decision theories, but also to the neuroeconomics research framework, which relies heavily on the translation of neurophysiological findings between human and non-human primate research. In fact, most non-human primate neurophysiological research relies on behavioural designs that share features of both description- and experience-based choices. As a consequence, it is unclear whether the neural mechanisms built from non-human primate electrophysiology should be linked to description-based or experience-based decision-making processes. The picture is further complicated by additional methodological gaps between human and non-human primate neuroscience research. After analysing these methodological challenges, we conclude proposing new lines of research to address them.

This article is part of the theme issue ‘Existence and prevalence of economic behaviours among non-human primates’.

## 1. The neuroeconomic research programme

The expected utility model was established as the standard normative model of decision-making under risk [1,2]. Integrating Bernoulli's intuition about the curvature of the utility function and probability theories, von Neumann and Morgenstern demonstrated that choices based on the expected utility (i.e. the product between the utility of an outcome and its probability) satisfies four basic axioms of rationality (completeness, transitivity, continuity and independence). Historically, the neoclassical economics research programme disregarded the study of the internal processes governing economic behaviours. Keynes' animal spirits [3] were considered unmeasurable, and economic theory was built on the assumption that the human mind as well the brain were ultimately black boxes. The ‘as-if’ hypothesis [4] illustrates this position by endorsing an instrumentalist epistemology: theory predictive power prevails on the realism of its initial assumptions. Accordingly, it was considered acceptable to rely on unrealistic assumptions regarding the unbounded cognitive capacities or perfect knowledge of economic agents, as far as the predictions were sufficiently accurate.

However, with the accumulation of behavioural evidence against the standard normative expected utility model, it soon appeared that it had to be profoundly amended to successfully account for actual decisions under risk [5,6]. Positive, descriptive, models of decision-making under risk that integrate insights from psychology, such as the notion of bounded rationality (i.e. humans display limited computational capacities), heuristics (taking computational *shortcuts* to make decisions) and biases (systematically distorted representations of behavioural variables) were then proposed and formalized [7–9]. Among the descriptive theories of decision under risk and uncertainty, ‘prospect theory’ (PT) had a strong empirical ground and stood out [8,10]. PT postulates that expected utility is calculated relative to a reference point (the *frame*), an asymmetric treatment of gains and losses (*loss aversion*), as well as a subjective weighting of probabilities (*probability distortion*). PT successfully explained known paradoxes (such as the Allais's paradoxes) and new ones (e.g. the Asian disease paradox, as well as a certain number of ‘real life’ irrational behaviours [11,12]).

However, despite these successes, some aspects of the descriptive approach, in general, and PT, in particular, remained unsatisfactory. First, it remained difficult to ultimately arbitrate between competing descriptive theories solely based on behavioural data. For instance, alternative behavioural theories have been proposed (such as rank-dependent utility, regret and disappointment theories; see [13] for a review) that make overlapping predictions with PT, making them hard to disentangle. Second, while making accurate predictions, PT, and other descriptive theories, do not specify which are the actual cognitive operations and how they are implemented by the brain. In terms of the Marrian analysis of modelling, PT (as other descriptive theories) is situated at the *computational* level that specifies which is the goal of the agent (in this case: maximizing a subjective utility that includes reference point dependence, loss aversion and probability deformation), but is silent concerning the *algorithmic* (i.e. what are the operations involved in the manipulation of decision variables) and *implementational* levels (i.e. how these operations are physically embodied and realized) [14].

A couple of decades later the time was ripe for a group of scholars of diverse origins to seek in neuroscientific data the way to overcome the limitations of descriptive theories, developed by psychologists and behavioural economists. This was facilitated by the rapid development of non-invasive neuroimaging techniques in humans (most notably functional magnetic resonance imaging: fMRI [15–17]) and improvement of single-unit electrophysiological recordings in monkeys [18,19]. The hope was (and still is) that, taking advantage of neuroscientific methods and concepts, *neuroeconomics* (as this raising field was named), would be able to address the epistemological issues of economic theories highlighted above.

Concerning adjudicating on competing theories (our first issue), by opening the brain ‘black box’ functional neuroimaging studies would provide an additional crucial observable measure—blood oxygen level dependent signal (BOLD: an aggregate and indirect measure of neural electrical activity), to compare, falsify and ultimately refine behavioural models. We define this approach as the *weak neuroeconomic agenda*, as it does not involve rewriting economic descriptive theories [20–22]. Coming back to our example, while making similar behavioural predictions in respect of preferences under risk, different theories postulate different utility functions that can be searched in the brain [23–25]. Assuming one knows where to look for utility representation in the brain,^1^ it would be, in principle, possible to assess which model better predicts its activity (a sort of neural model comparison: see [29]). Beyond comparing different theories, the neural activity could in principle help refining a theory by fixing some of its parameters. For instance, in many circumstances, PT is silent about how the reference point should be set [30]. Assuming one knows where to look for positive (gain) and negative (loss) utility representations in the brain, in some cases the reference point could be inferred comparing the profile of activity of the ‘gains’ and ‘losses’ areas^2^ [25,33].

Concerning building new theories (second issue), accepting the fundamental ontological tenet that (economic) decisions ultimately stem from neural activity in the brain (which is a standard materialistic and monistic solution to the mind-body problem, see [34]), entails that neuroscientific methods should provide the conceptual and methodological tools necessary to develop new, neurobiologically grounded, *neural* models encompassing the algorithmic and implementational levels. By contrast with the previous approach, we define this approach as the *strong neuroeconomic agenda*, as it involves rewriting economic theories in neurobiological terms. By integrating biological constraints and cost functions, these hypothetical neurobiologically grounded economic models have the potential of explaining why human decision-making presents certain biases from a biologically (not logically or statistically) normative perspective [35,36].

The methodological requirements of the two main neuroeconomics agenda are not quite the same. The weak neuroeconomic agenda can, in principle, be fulfilled by experiments relying on aggregate and indirect measures of the neural activity, such as the BOLD signal recorded by fMRI scanners in areas encoding subjective values. Furthermore, since the goal is arbitrating between different behavioural theories of decision-making developed by psychologists and economists, the experiments belonging to this research agenda should be preferentially (if not exclusively) performed in humans.

On the other side, as neural models are, ultimately, models of which information is encoded in neurons and how neurons are connected (networks), the strong neuroeconomic agenda research programme cannot be pursued only relying on fMRI neural signals.^3^ In fact, BOLD signal, at its best resolution, aggregates over thousands of neurons [37–39]. Furthermore, it is still unclear to which extent it reflects presynaptic or postsynaptic activity (probably a mixture of both) [39,40]. Such neural models should eventually be validated based on the recording of single-cell activities, which is, for obvious ethical reasons, nearly impossible in humans.^4^ This is why neuroeconomics research, from its very inception, strongly relies on electrophysiological research on animal models, which have been employed in the study of neural mechanisms and cognition for almost 80 years [42]. Monkeys (especially rhesus monkey: *Macaca mulatta*), are particularly popular models, because they present a wide behavioural repertoire and high degree of neuro-anatomical homology with humans, especially concerning the prefrontal cortices that underpin decision-making [43].

**Figure 1.**
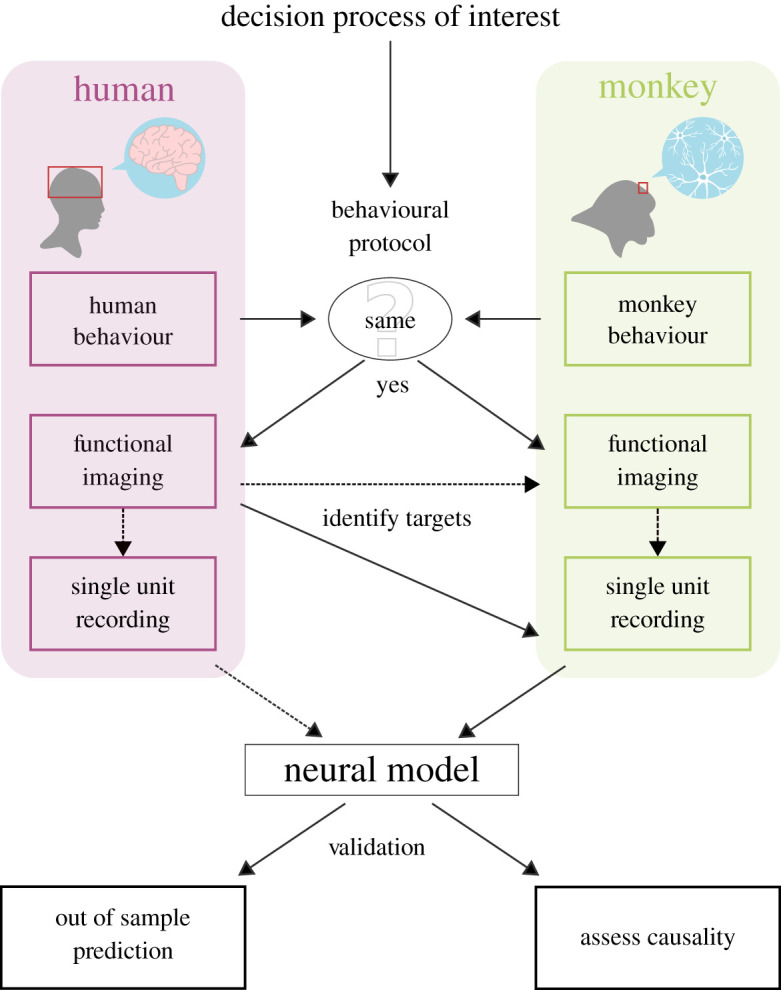
Prototypical workflow combining human (purple) and monkey (green) data to pursue the strong neuroeconomic agenda. Dotted lines designate optional steps. (Online version in colour.)

In figure 1, we represent what a prototypical workflow should look like to combine human and monkey data to deliver a neural model of decision-making. Of note, we describe it from an abstract perspective of theory-building, but in reality, its different steps can occur simultaneously (or in reverse order), and in very distant laboratories. Once identified as a behavioural process of interest (e.g. decision-making under uncertainty), a behavioural protocol is designed (typically, a series of choice problems involving different amounts of rewards and probabilities) and administered to both humans and monkeys. If the behaviour is comparable across species (meaning that the monkey represents a valid *experimental model* of human behaviour^5^), functional imaging in humans can then be deployed to identify neural targets encoding macroscopic variables (e.g. probabilities, outcomes) that are later used to guide the selection of the areas where neurons will be recorded in monkeys. A desirable intermediate step, to reinforce the functional correspondence between human and monkey brain activations, would be to also deploy fMRI in monkeys [45]. Similarly, in some neurologic and psychiatric diseases, intra-cranial neural activity can also be recorded in humans [41]. Finally, all these data can then be combined together to propose and validate a neurobiologically plausible model of the behavioural process of interest. Thereafter, the proposed model should be validated using lesions and assessing its generalizability. Methods such as trans-cranial magnetic stimulation and brain lesions can be used to test the alleged causal relationship between neural correlates and behavioural processes [46–48]. The model's ability to generalize can be assessed by generating predictions in tasks involving different decision problems and behavioural processes (out-of-sample validation).

A crucial step in this workflow is checking that humans and monkeys display the *same* behavioural processes and biases as a result of a true homology. This is something notoriously tricky to assess, because several, to some extent unavoidable, methodological differences exist between human and non-human primate research.

**Figure 2.**
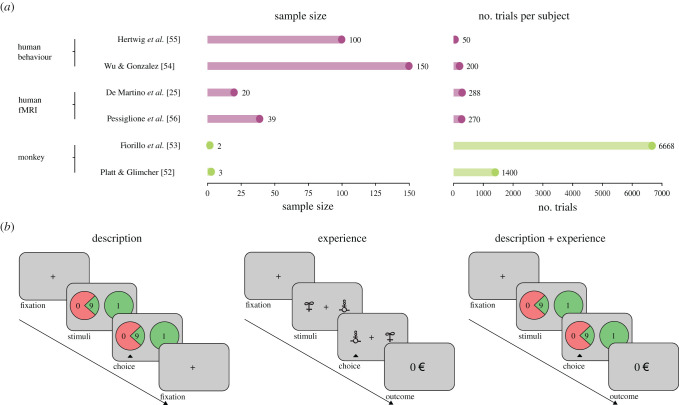
Methodological differences between description, experience and description–experience studies. (*a*) Sample size and number of trials listed in two electrophysiological studies [52,53], two human fMRI studies [25,56] and two human behavioural studies [54,55]. (*b*) Successive screens of a trial in the different behavioural decision-making paradigms. In pure 'description' paradigms, decision variables are explicitly described and no feedback is provided. In pure 'experience' paradigms, decision variables are hidden and feedback is provided on a trial-by-trial basis. In the ‘description plus experience’ paradigms, decision variables are explicitly described and feedback is provided on a trial-by-trial basis. (Online version in colour.)

The foundational experimental paradigm of behavioural decision-making research consists in making choices between ‘lotteries’ or ‘gambles’, i.e. options associated with known or unknown probabilities of obtaining different outcomes [2,5]. According to the gambling metaphor of individual choice [49], lotteries are believed to be prototypical of real-life decisions [50]. Outcomes and their probabilities are described to participants, who often (especially in the first generation of behavioural economics studies) make only one or very few decisions, without being informed about the outcome of their choices (in general to purposely prevent learning processes from influencing decision-making [51]). On the other side, monkey electrophysiological research adopts very different methodological standards. For various reasons (including ethical ones), monkey studies are limited in terms of sample size, and consequently, the number of observations per subject is greatly increased in order to increase statistical power and reduce measurement noise. In fact, behavioural tasks in monkeys display a greater number of trials per subject, collected on a sample size of often less than five subjects (e.g. [52,53]). Both parameters (sample size and number of trials) are roughly a couple of orders of magnitude different compared to what is common practice in behavioural economics (e.g. [54,55]) (figure 2*a*). Interestingly, fMRI studies of decision-making present experimental parameters somehow in-between those used in monkeys and human studies: they usually involve hundreds of trials and also sample sizes of about 20–40 subjects (see two notable examples in neuroeconomics: [25,56]). Assuming that decision-making possesses ergodicity (i.e. the behaviour averaged across trials is the same as the behaviour averaged across subjects), different ratio trial/participants *per se* should not present a big challenge to compare results from human and monkey studies (but note that ergodicity does not seem to be granted for psychological processes, see [57]). However, in addition to these quantitative differences, in monkey studies, an outcome (usually a primary *reward*) is provided on a trial-by-trial basis. This is because a monkey would simply stop doing the experiment in the absence of extrinsic motivation. Thus, in virtually all cases monkey experiments include a *reinforcement learning* component, where actions are associated with past outcomes. This is true even when the paradigm involves establishing a symbolic system to communicate outcomes and probabilities. In fact, in the absence of a shared language or semantic system to communicate, monkeys are compelled to learn any representational system by trial-and-error from feedback.

In the present article, we argue that the above-mentioned differences do not only present a *technical issue*, but also a major *epistemological challenge* for the (strong) neuroeconomic agenda. We detail why below.

## 2. The experience–description gap

As mentioned before, foundational contributions to behavioural decision-making research were made through the use of explicitly described gambles. Several representations have been used to convey outcome values and probabilities, including textual and numerical descriptions (e.g. [5,8,54]), later replaced by visual cues such as pie-charts (e.g. [25,58]). In these paradigms, the information pertaining to the decision-relevant variables is processed by verbal and mental calculation systems and relies upon some degree of semantic knowledge to decode the meaning of the symbols used. In addition to that, decision problems were usually presented only once and, in case multiple decision problems were used, the final outcome (i.e. the realization of the lottery) was usually not displayed on a trial-by-trial basis (figure 2*b*).

**Figure 3.**
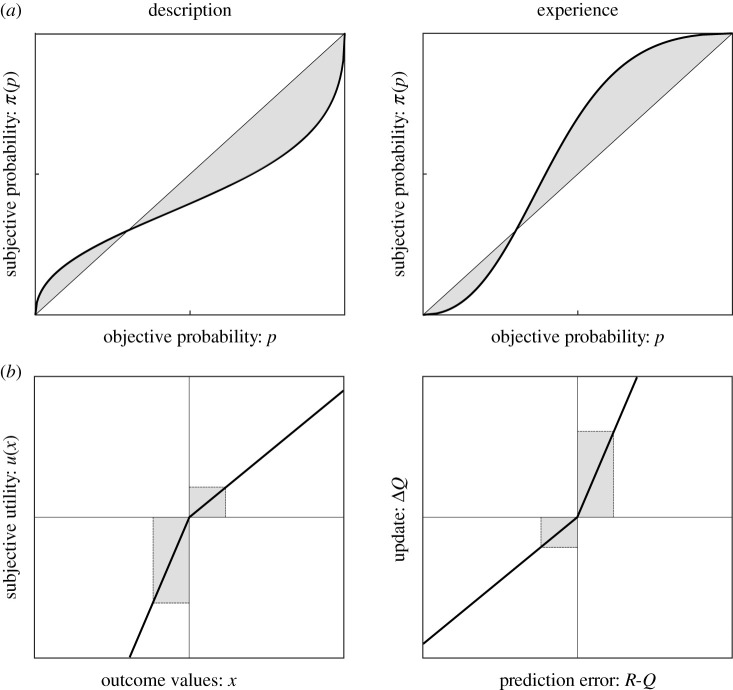
(*a*) Illustration of the nonlinear transformation of probabilities in description (left panel) and experience (right panel). In the description domain, subjective probability is reflected by a probability weighting function (here denoted *π*) following an inverse S-shape (i.e. low probabilities are overweighted while high probabilities are underweighted). This tendency is reversed when it comes to the experience domain, where the curve follows an S-shape. (*b*) Illustration of the classical linear utility function in the description domain (left panel) and the update of the value function for the experience domain (right panel). In description, the utility curve displays a steeper slope for losses than gains. In experience, an opposite phenomenon is frequently observed. The sign of the prediction error (i.e. the difference between the obtained reward *R* and the associative value *Q*) affects the learning rate.

However, relatively few situations in real life match the characteristics of the pure *description-based* paradigms, namely complete and explicit information about outcome values and probabilities. In fact, in many circumstances, it seems rather prudent to assume that information about outcome values and probabilities are shaped by past encounters of the same decision problem. Experimentally, this configuration is often translated into *multi-armed bandit* problems (starting with Thompson [59], but see [60] for a review), where the decision-maker faces abstract cues of unknown value and has to figure by trial-and-error the value of the options. Computationally, behaviour in multi-armed bandit problems is generally well-captured by associative or reinforcement learning processes [61]. In the early 2000s, a line of enquiry arose where researcher translated the typical decision problems used in behavioural economics (i.e. involving choices between a safe and a risky prospect in the gain and loss domain^6^) into experience-based paradigms [55,63,64] (figure 2*b*). Systematic comparisons between these two decision-making modes revealed the existence of robust *description–experience gaps* regarding risk preferences in humans [65–67] . More precisely, probability weighting functions eventually show opposite deformations when comparing description-based and experience-based choices (figure 3, box 1). In particular, most of the tenets of PT do not seem to hold in experience-based choices [8]. While traditionally, in the description domain, the occurrence of rare events is overestimated (*possibility* effect) and the occurrence of frequent events is underestimated, experience-based decisions tend to show the opposite biases: an effect that is only partially explained by incomplete sampling [55,63,64,66].

Box 1. **Description- and experience-based behavioural models.**

In this box, we sketch the formalisms standardly employed to explain and quantify risk preferences in description-based and experience-based decisions. Description and experience paradigms radically differ in how they model decision under risk. In the description domain, risk preferences are the *direct* result of subjective deformations of probabilities and outcomes that are explicitly stated. On the other side, in the experience domain there is no separate representation of outcomes' probabilities and no explicit deformation of outcomes’ values. Consequently, risk preferences are the *indirect* result of the learning process that links past outcome information to subsequent choices. Eventually, these two approaches lead to different explanations of risk attitudes.

Risk preferences in description-based paradigms are commonly explained by prospect theory (PT). The expected value of *k* iterations of the same gamble *X* (which is random variable) is computed as follows:

$$E(X)=\overset{k}{\underset{i=1}{\sum }}p_{i}x_{i},$$

where *x_i_* is the value of an individual outcome and *p_i_* is the objective probability of the outcome. PT states that the utility of an outcome, that is the subjective value *u*(*x_i_*), is nonlinear and modulated by different parameters: *α* and *β*, that are the power to which, respectively, a positive or negative outcome are elevated, and *λ* the loss aversion coefficient. Thus, the PT utility function is defined as follows:

$u(x_{i})=\{\begin{array}{c} x_{i}^{\alpha }\;\;\;\;\;\;\;\;\;\;\;\;\;\;\;\;\;\;\text{if}\;x_{i}\geq 0\\ -\lambda {\left(-x_{i}\right)}^{\beta }\;\;\;\;\;\text{if}\;x_{i}<0 \end{array},$

an *α* ≤ 1 corresponds to risk aversion in the gain domain (the intuition dates back to Bernoulli), *α* > 1 corresponds to risk-seeking behaviours. In the loss domain, the same relation is true concerning the values of *β*. A value of *λ* *>* 1 corresponds to loss aversion; its typical empirical value is around 2 [10,68]. A decision-maker with *α* < 1, *β* > 1 and *λ* *>* 1 will present different risk preference in the gain (risk aversion) and the loss (risk seeking) domain (figure 3*b*).

In addition, PT postulates a subjective deformation of probabilities. There are multiple ways to mathematically express the probability weighting function. One of the most common is the ‘Prelec’ function [69]:

$$\pi (\;p_{i})=e^{-\delta {\left(-\log(\;p_{i})\right)}^{\gamma }}$$

with *δ* controlling the elevation and *γ* the curvature. When both parameters are set to 1, the function tends to linearity. The more *γ* > 1, the more the function adopts an S-shape. A classical result is the overweighting of low probabilities compared to high probabilities, where the direction of the curve follows an inverse S-shape (figure 3*a*), with *γ* < 1. Note that another probability weighting function has been proposed [54]. Finally, the subjective expected utility is given by

$$\text{SEU}(X)=\overset{k}{\underset{i=1}{\sum }}\pi (\;p_{i})u(x_{i}).$$

By the variation of these parameters, PT accounts for inter-individual differences in risk preferences. Of note, concurrent theories such as regret theory [70] or rank-dependent utility models [71], which use very different representational structures and parameterizations, are also used to model decision-making under risk.

Experience-based paradigms can be seen as reinforcement learning problems operationalized as k-armed bandit tasks [61]. Consider an environment composed by a state vector *S*, with *s* ∈ *S*. In each of states *s*, there are available actions denoted *a* ∈ *A*. Each state-action pair has an underlying reward probability distribution, such that *P*[*R*|*s*, *a*], is the probability of obtaining the reward *R*, knowing the state-action couple (*s*, *a*). An agent must then follow a policy in order to maximize a state-action value function *Q*(*s*, *a*) (i.e. to maximize the average expected reward). A common learning policy is to compute subsequently to a choice of the prediction error *δ*, that will be used to incrementally update the value associated to a specific state-action pair (*s*, *a*):

δ=R−Q(s,a)

Q(s,a)←Q(s,a)+αδ

with *α* the learning rate that determines to what extent newly acquired information overrides the previous. In this paradigm, inter-individual variability in behaviours can be accounted for by differences in individual parameters such as the aforementioned learning rate *α*. However, this model with only one parameter is too simple to accommodate different risk preferences.

A way to refine this model to account for different risk preferences, is to allow for two different learning rates, *α*^+^ and *α*^−^:

$$Q(s,a)\leftarrow Q(s,a)+\{\begin{array}{c} \alpha^{+}\delta \;\;\;\;\text{if}\;\delta >0\\ \alpha^{-}\delta \;\;\;\;\text{if}\;\delta <0 \end{array}$$

If *α*^+^ = *α*^−^, the two learning rates model is equivalent to a one learning rate model. We define the tendency to preferentially update *Q*(*s*,*a*) from positive prediction errors rather than negative prediction errors as *positivity bias* (or *loss neglect*) (*α*^+^ *>* *α*^−^). Conversely, we define the opposite situation (*α*^+^ *<* *α*^−^) as *negativity bias* (or *loss enhancement*).

The learning rate asymmetry has direct consequence for risk preferences in the setting where a subject has to learn the value of a safe (say a fixed value of 0) and a risky (say 50% chance of winning/losing one euro) option. A subject displaying a positivity bias would neglect the past losses and will, therefore, be a risk-seeker (figure 3*b*). Conversely, the negativity bias implies risk aversion. Both pessimistic and optimistic biases have been reported in the literature, with the latter bias being more frequently reported [72–75].

While it is tempting to see the positivity bias as the experience-based antithesis of loss aversion, their formalism and psychological interpretations are quite different and they are, therefore, not mutually exclusive. Indeed, loss aversion concerns the valuation of *prospective* losses, while the positivity bias concerns the *retrospective* assessment of past losses.

It is important to note that, in humans, although the average values of the behavioural biases are reported as described above (for instance: inverse S-shape in description-based paradigms and loss neglect in experience-based paradigms; see figure 3*a*), their results are further tempered by a high degree of inter-individual variability in the bias parameters. At the individual level, some subjects may in fact display opposite biases in both experimental settings [72,76]. If inter-individual variability is equally high in other primates, the fact that monkey studies use very small sample size (figure 2) can contribute to explaining the comparably less consistent picture observed (table 1).

**Table 1.** Studies investigating risk attitudes in rhesus monkeys. E, experience-based paradigms (i.e. without explicit representation of outcomes and probabilities); D, description-based paradigms (i.e. involving explicit representation of outcomes and probabilities; note that in monkeys this implies a 'description plus experience' set-up); liquid, the utilization of either water or fruit juice; tokens, the acquisition of a secondary reward, which is later exchanged for a primary reward; seek, an overall preference for the risky option; avoid, an overall preference for the safe option; inverse S-shape, the probability distortion postulated by prospect theory; S-shape, the probability distortion traditionally found in experience-based paradigms; N/A, the information is not available.

study	sample size	modality	reward	risk attitude in gains	risk attitude in losses	probability distortion	loss aversion
McCoy & Platt [80]	2	E	liquid	seek	N/A	N/A	N/A
Hayden & Platt [81]	2	E	liquid	seek	N/A	N/A	N/A
Hayden *et al*. [82]	5	E	liquid	seek	N/A	N/A	N/A
Long [83]	3	E	liquid	seek	N/A	N/A	N/A
Watson [84]	8	E	liquid	seek	N/A	N/A	N/A
O'Neill & Schultz [85]	2	E	liquid	seek	N/A	N/A	N/A
Heilbronner *et al*. [86]	3	E	liquid	seek	N/A	N/A	N/A
Kim *et al*. [87]	2	E	liquid	seek	N/A	N/A	N/A
Heilbronner & Hayden [88]	2	E	liquid	seek	N/A	N/A	N/A
Xu & Kralik [89]	2	E	liquid	seek	N/A	N/A	N/A
Smith *et al*. [90]	7	E	liquid	seek	seek	N/A	N/A
Hayden *et al*. [91]	4	D	liquid	seek	N/A	N/A	N/A
So & Stuphorn [92]	2	D	liquid	seek	N/A	N/A	N/A
Yamada *et al*. [93]	?	D	liquid	avoid	N/A	N/A	N/A
Raghuraman & Padoa-Schioppa [94]	2	D	liquid	seek	N/A	N/A	N/A
Staufer *et al*. [95]	2	D	liquid	seek	seek	inverse S-shape	N/A
Farashahi *et al*. [96], experiment 1	3	D	liquid	seek	N/A	none	N/A
Farashahi *et al*. [96], experiment 2	3	D	token	seek	seek	S-shape	N/A
Chen & Stuphorn [97]	2	D	liquid	seek	seek	inverse S-shape	N/A
Nioche *et al*. [98]	2	D	liquid	avoid	seek	inverse S-shape	yes
Ferrani-Toniolo *et al*. [99] experiment 1	2	D	liquid	N/A	N/A	inverse S-shape	N/A
Ferrani-Toniolo *et al*. [99], experiment 2	2	D	liquid	N/A	N/A	S-shape	N/A
Eisenreich *et al*. [100]	3	D	liquid	seek	seek	N/A	N/A

In description-based choices, a behavioural hallmark of loss aversion (overweighting of negative outcomes) is the *reflection effect*, where subjects are risk averse in the gain domain and risk seeking in the loss domain. The opposite pattern has been repeatedly found in the experience-based decisions [67]. This observation may be explained by biases in the learning process, such as remembering preferentially extreme outcomes or integrating preferentially better-than-expected outcomes [72,77]. Finally, a smaller subset of studies investigated a hybrid situation where decision problems are fully described, choices are repeated and followed by a trial-by-trial feedback. These ‘*description plus experience*’ paradigms showed that probability distortions compatible with prospect theory are initially present, but corrected by the presence of feedback [78,79]. To summarize, the whole spectrum of decision-making under uncertainty in humans is far from being fully captured by PT's loss aversion and subjective probability deformation. Specifically, different descriptive models seem to apply as a function of how outcome and probability information is conveyed. In what remains of the paper, we illustrate why we believe that this feature seriously challenges leveraging on neural and behavioural data in monkeys to build a neural model of decision-making under uncertainty.

## 3. Decision under risk in monkeys

In this section, we try to address the question of whether monkeys are a good experimental model for human decision-making under uncertainty. We will focus this survey on rhesus monkey (*Macaca mulatta*) results because most electrophysiological studies are performed in this species (but see [44] for a more detailed review including other primates). Asking whether monkeys are a good experimental model translates into asking whether in the laboratory setting their behaviour displays the distinctive features and biases observed in humans. We stress again that the comparison is complicated by the fact that *pure* description-based paradigms cannot exist in monkey studies because of the lack of language. In fact, in monkey studies, whenever outcomes and probabilities are conveyed via a symbolic system, the system is nonetheless learned and maintained by trial-by-trial outcomes (i.e. a situation similar to the ‘*description plus experience*’ paradigm, described above). In such ‘pseudo’ description-based paradigm, monkeys are trained to associate continuous variations in one visual feature (e.g. colour or size) to continuous variations of a decision variable (e.g. outcomes or probabilities). The comparison is further complicated by the fact that only few studies formalize risk preferences in terms of model parameters (such as probability distortion, loss aversion or learning rates) and data reporting is often limited to behavioural measures.

The general picture (table 1) emerging from ‘pseudo’ description-based paradigms in monkeys (i.e. studies relying on learned symbolic systems to communicate values) is, at best, mixed. PT has been explicitly tested in paradigms using visual cues carrying symbolic information similar to those presented to humans (e.g. pie-charts). Only a few studies show results in conformity with the pattern of description-based decisions observed in humans. Risk aversion, suggestive of marginally decreasing utility in the gain domain, has been rarely reported [93]. Nioche *et al*. [98] is the sole study confirming all PT features: marginally decreasing utility (risk aversion in the gain domain), loss aversion (risk seeking in the loss domain) and subjective probability weighting consistent with overestimation of rare events. Probability weighting function consistent with standard PT has been reported by other studies, but the same studies also reported increasing marginal utility and risk seeking in the gain domain, which is not typically observed in description-based decisions in humans [95,97]. Many others pseudo description-based experiments also reported risk-seeking attitudes and/or marginally increasing utility in gains [91,92,94,96]. In addition, although the traditional inverse probability weighting function has sometimes been observed [95,98], variation of experimental design features (such as randomly mixing gambles instead of repeating the same gambles sequentially) can reverse the direction of the probability weighting function [99].

Regarding ‘pure’ experience-based studies in monkeys (i.e. involving no symbolic system to communicate values), the picture is somehow clearer. Indeed, rhesus macaques exhibit robust risk-seeking behaviour in the gain domain [80–89]. Risk-seeking attitudes have also been reported in the loss domain [90].

Risk-seeking behaviour in experience-based studies can be computationally explained by an increased sensitivity to positive (compared to negative) prediction errors (‘positivity’ bias) which is generally documented in human reinforcement learning (box 1) [72–74]. This hypothesis is corroborated by studies demonstrating a stronger impact of past positive outcome in choices using either model-free or model-based measures [81,82,101].

Finally, it can be argued that if monkeys are a good model for human decision-making under uncertainty, they should display a description–experience gap. To our knowledge, so far only one study explicitly tackled this issue [102]. Monkeys were asked to make repeated choices between safe, and risky options, whose outcome probability was either learned by experience or described by the ratio between colours on a rectangle. Replicating previous findings in monkeys, and in discordance with the standard result in humans, Heilbronner and Hayden found that monkeys were risk-seekers in the description domain. However, consistent with the gap observed in humans, they also found that risk-seeking behaviour was higher for experience-based cues.

To summarize, the literature seems to suggest that monkeys' decision-making for experience-based choice is quite consistent with what is observed in humans in terms of risk preference. This is consistent with a large body of literature showing that the neural substrates of reinforcement learning are largely preserved in the two species [103,104]. Risk seeking in this context may be driven by a higher learning rate from positive compared to negative prediction errors, which is essentially a computational reinforcement learning translation of the ‘hot hand’ fallacy [105,106]. The situation is much less reassuring concerning description-based decisions, as preferences compatible with PT are rarely observed. This can be due to the fact that pseudo description-based design in monkeys resembles the ‘description plus experience’ set-up in humans, where PT-like deformations are blunted or even disappear, as if description-based and experience-based biases reciprocally cancel themselves [78,79]. As a result, it remains unclear to what extent description-based processes can be elicited in the non-human primate animal model.

## 4. The impact of other experimental differences

**Figure 4.**
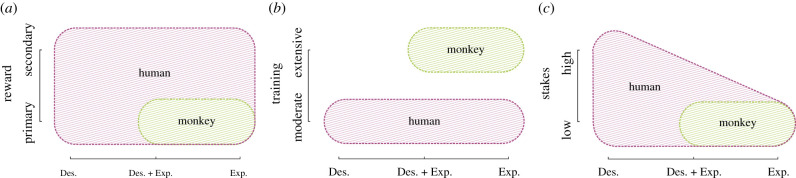
The figure illustrates how human (purple) and monkey (green) experimental settings map into a four-dimensional space, whose axes are: the way value information is provided (from description to experience); the nature of the reward (from primary to secondary; *a*), the amount of training (from moderate to extreme; *b*) and the level of the stakes (from low to high; *c*). (Online version in colour.)

Experimental results concerning decision-making under uncertainty in monkeys do not seem to straightforwardly comply with the predictions of PT. Overall it seems that monkeys' behaviour is better accounted for as an experience-based decision process, which is consistent with the fact that pure description-based paradigms are not possible and monkey experiments always involve trial-by-trial feedback. The systematic presence of trial-by-trial feedback is not the only systematic methodological difference between the monkey and human studies (figures 2 and 4).

First, monkey studies essentially rely on primary rewards (mainly water or fruit juice), while human studies are realized mainly with secondary rewards (sometimes hypothetical ones) and primary reinforcers are only occasionally used [107,108]. Preliminary evidence from a study comparing risk propensity for different kinds of rewards in humans (money versus sport beverage) and monkeys showed similar patterns in the two species, thus suggesting that in more comparable experimental condition risk preferences in both species could converge [109]. Furthermore, while the neural correlates of different kinds of rewards converge in the ventral prefrontal and striatal systems (principle of the common currency; [110]) they also have specific correlates, which may contribute to the different neural mechanisms and result in distinct, reward-specific, risk preferences [107]. On the other side, a proxy for secondary reward can be found in monkey paradigms that involve collecting (virtual) tokens to be later exchanged for a primary reward. Unlike pure primary reward tasks, where losses cannot be implemented (it is impossible to take some fruit juice away from the stomach of a monkey), tokens have the advantage of making possible subtracting previously acquired rewards from the animal, thus inducing ‘losses’ in the same manner as in human. However, a recent study using tokens, showed risk-seeking attitudes comparable to that observed using primary reward [96]. Furthermore, when tokens are used, they are almost immediately changed against primary reward, making them not really comparable to money, whose value is much more permanent. Taken together, the available evidence suggests that the primary/secondary reward dichotomy does not explain the fact that human description-based biases are hardly observed in monkeys.

Second, in addition to the difference in the nature of the reward, description-based paradigms in humans and paradigms in monkeys often present a systematic difference in the amount of the reward (figure 4). Indeed, most of the original studies about PT used hypothetical gambles of hundreds of dollars and the same biases have been replicated using real stakes of about a month's salary [111]. On the other side, monkey studies use very small amounts of rewards (mere drops of liquids). It has been argued that part of the description–experience gap may simply derive from this difference in stake instead of being induced by fundamental differences in the decision-making process [88]. This would be consistent with Markowitz utility function which supposes risk seeking for small stakes (peanuts effect) before converting to risk aversion for higher stakes [112] and is supported by the finding that increasing the relative amount of reward (by reducing its frequency) decreases risk seeking down to risk neutrality in monkeys [88,112]. However, risk aversion in the gain domain (and a reverse pattern in the loss domain: the reflection effect) has also been observed with small stakes in description-based decisions in humans [67]. Thus, available evidence suggests that differences in the size of the stake cannot *fully* explain the fact that human description-based preferences are hardly observed in monkeys.

Finally, another notable difference between human and monkey experiments is represented by the amount and the type of training required to perform the task (see figures 2 and 4). In human experiments, task training rarely takes more than a few minutes (in some extreme cases of description-based paradigms, there is virtually no training: subjects are just *asked* to reveal their preferences). On the other side, monkey experiments require extensive training, in general spanning several months (usually training takes longer than the experiment itself). It can be, therefore, argued that their behaviour becomes to some extent habitual or automatized: a cognitive state that contrasts dramatically with the declarative and deliberative stance of description-based choices taken by humans [113]. In addition to that, training in monkeys (and other animals) often involves simplified versions of the task (often deterministic contingencies), which may reinforce specific risk preferences. Although the role of extended (several days, weeks) training and the resulting behavioural automation (or habituation) in risk preferences is unclear, it may contribute to the fact that human description-based biases are rarely observed in monkeys.

## 5. Conclusion and perspectives

Our review suggests that the rhesus monkey is a *partial* model of human decision-making under uncertainty. Risk preferences in monkeys are generally better explained as experience-based processes. Accordingly, monkeys proved to be a very good model of human reinforcement learning processes, providing crucial insights into its neural implementation (the dopamine prediction error hypothesis: [56,62,114]). The situation is less clear concerning description-based choices. In paradigms using explicit symbolic information about decision variables, monkeys only rarely displayed risk preferences compatible with human results. Deciding by description implies a symbolic system of communication. While in humans this system pre-exists (language), in monkeys it has to be learnt by trial-and-error, thus irremediably confounding description and experience. In addition to differences in the way value information is conveyed (experience- or description-based), other methodological factors (training, reward type and stakes) further drive apart the experimental set-ups of the two species. This situation is problematic as building a neural model of decision-making under uncertainty should integrate human (fMRI) and monkey (single unit) neurophysiological data, while explaining risk preferences in a wide range of situations that span from pure description-based choices to pure experience-based choices.

We propose further lines of research that could eventually help filling these gaps and ultimately fulfilling the strong neuroeconomic agenda. On the human side, the description–experience gap has been extensively studied at the behavioural level, but surprisingly neglected at the neural level. A notable exception [115], found different neural representations for description- and experience-oriented decisions. Furthering this line of enquiry would prove useful to redefine the target areas to look *specifically* for description-based processes in monkey electrophysiological studies.

With the development of online testing techniques, it is becoming easier to implement extended massive training in humans [116]. Translated in the field of decision-making under risk, these experiments would provide crucial insights into the impact of extensive training in risk preferences. While, description-based studies in monkeys require learning *ex novo* a symbolic system, in humans the meaning of pie-charts is provided by the language. It would be interesting to put humans in situations where they have to figure out by trial-and-error the code linking continuous visual features to decision variables.

In general, all the efforts aimed at increasing the methodological overlap between human and monkey studies will provide further insights into what are the behavioural processes shared across the two species. Popularizing fMRI experiments in monkeys would help confirm the neuro-anatomical targets and increase the focus on shared neural systems. The token paradigm (conceptually closer to the notion of the secondary reward) offers the possibility to implement losses in monkeys, hence facilitating the cross-species study of loss aversion.

Finally, on the monkey side, PT has been sporadically replicated. It will be important to clarify and formalize the experimental factors (in terms of stimuli, training and reward type; see table 1) that predict whether PT-like behaviour will be observed in a monkey experiment [88]. Determining under which experimental conditions PT is replicated in monkeys will imply a deeper understanding of the cognitive mechanisms underlying decision-making under uncertainty.

## Endnotes

1 Subjective utility (or subjective value) representation seems to be distributed across a network of areas that include the ventral and the dorsal prefrontal cortices (both medial and lateral part), posterior cingulate cortex, the striatum, the insula, the amygdala and the hippocampus [26–28].

2 It is indeed the case that brain systems encoding positive and negative values are, at least partially, dissociable. Losses are generally encoded by the insula, the amygdala and the dorsal prefrontal cortex, while gains are generally encoded in the ventral prefrontal and the striatum [31,32].

3 Other non-invasive imaging techniques, such as magneto- and electro-encephalography present no advantage over fMRI when it comes to inferring single unit activity. They present better temporal resolution traded off against a worst spatial resolution.

4 There are a few exceptions of single unit recordings in humans, obtained from neurologic patients undergoing brain surgery. While informative, these data are limited by the fact the neuro-anatomical targets cannot be chosen freely and that findings may not generalize to the general population [41].

5 Of course, there is a lot of information to be gained also in the case where humans and monkeys do not display the same decisions and biases. Such differences currently represent a strong area of research in comparative psychology and ethology [44]. However, the (not so implicit) assumption of the vast majority of research in neuro-economics is that monkeys are valid experimental models for human cognition, and they are not investigated for comparative reasons.

6 In the human reinforcement learning literature, the most frequently used paradigms involve options that possess, at a given trial, different expected values but overall similar risk level [56,62]. As a result the human reinforcement learning literature is more concerned about measures of objective performance rather than subjective preference.

## Data accessibility

This article has no additional data.
